# A Full-Genomic Sequence-Verified Protein-Coding Gene Collection for *Francisella tularensis*

**DOI:** 10.1371/journal.pone.0000577

**Published:** 2007-06-27

**Authors:** Tal Murthy, Andreas Rolfs, Yanhui Hu, Zhenwei Shi, Jacob Raphael, Donna Moreira, Fontina Kelley, Seamus McCarron, Daniel Jepson, Elena Taycher, Dongmei Zuo, Stephanie E. Mohr, Mauricio Fernandez, Leonardo Brizuela, Joshua LaBaer

**Affiliations:** 1 Harvard Institute of Proteomics, Department of Biological Chemistry and Molecular Pharmacology, Harvard Medical School, Cambridge, Massachusetts, United States of America; 2 DF/HCC DNA Resource Core, Harvard Medical School, Cambridge, Massachusetts, United States of America; Baylor College of Medicine, United States of America

## Abstract

The rapid development of new technologies for the high throughput (HT) study of proteins has increased the demand for comprehensive plasmid clone resources that support protein expression. These clones must be full-length, sequence-verified and in a flexible format. The generation of these resources requires automated pipelines supported by software management systems. Although the availability of clone resources is growing, current collections are either not complete or not fully sequence-verified. We report an automated pipeline, supported by several software applications that enabled the construction of the first comprehensive sequence-verified plasmid clone resource for more than 96% of protein coding sequences of the genome of *F. tularensis*, a highly virulent human pathogen and the causative agent of tularemia. This clone resource was applied to a HT protein purification pipeline successfully producing recombinant proteins for 72% of the genes. These methods and resources represent significant technological steps towards exploiting the genomic information of *F. tularensis* in discovery applications.

## Introduction

Over the past several years, complete or nearly complete sets of clones representing the open reading frames (ORFs) of various species have been constructed and made available [1]–[Bibr pone.0000577-LaBaer1] [Bibr pone.0000577-Rual1] [Bibr pone.0000577-Park1] [Bibr pone.0000577-Ecker1] [Bibr pone.0000577-Matsuyama1] [7]. These collections often employ recombinational cloning vectors, enabling the transfer of the ORFs into virtually any protein expression vector in a simple conservative transfer reaction. Once transferred, these expression clones can be employed in a wide variety of assays, including high-throughput (HT) cell-based and proteomic discovery assays [2], [4], [8]–[Bibr pone.0000577-Reboul1] [Bibr pone.0000577-Parrish1] [Bibr pone.0000577-Temple1] [12] .

Clone collections have been used successfully to produce proteins using *in vitro*, bacterial or insect cell expression systems [13], [14]. Although several heterologous protein expression systems are capable of HT protein expression, simplicity and ease of handling have made the bacterial systems the best starting point to express large numbers of recombinant proteins [15], [16].

Among the most important properties that transferable clone collections should embody include comprehensive genomic representation of the ORFs and full length sequence validation, a combination of features that has thus far eluded the collections available today. In part, this is because sequence validation of clones is a tedious process that cannot be easily achieved without a well developed automated pipeline. Nevertheless, the major use of these collections will be to study protein function, emphasizing the critical importance of full length sequence validation.

There is a pressing need to generate clone and protein resources for highly infectious organisms that could be used in bioterrorism. One such organism is *Francisella tularensis*, a highly virulent, gram-negative, facultative intracellular pathogen that is the causative agent of tularemia. *F. tularensis* is capable of infecting many mammalian species and cell types, and has been isolated from more than 250 animal species, including mammals, arthropods and protozoa [17]. In mammalian hosts, *F. tularensis* thrives in the intracellular environment of macrophages [18]. The virulent subspecies *F. tularensis tularensis* is found in North America. As few as ten cells of this subspecies are sufficient to cause an infection in humans and 30% to 60% of untreated infections are fatal [19]. The high infection capability and transmission of the organism by aerosols pose a significant threat, leading the U.S. Center for Disease Control (CDC) to consider *F. tularensis* a category A biodefense pathogen [19]. Recent studies on the pathogen have focused on genomic analysis, identification of antigen targets for vaccine development, and on understanding the mechanisms of infection [20], [21]. The genome sequence of *F. tularensis* (subsp*. tularensis SCHU S4*) was published in 2005 [20]. The organism has a genome of approximately 1.9 MB that is AT rich (33% GC content) and is predicted to encode 1,804 genes, of which 302 sequences are unique to *Francisella* [20]. More than 10% of genes are predicted to be pseudogenes or gene fragments [20]. The current annotation at NCBI includes 1,852 genes, of which 1,603 represent putative protein-coding sequences.

The study of *F. tularensis* pathogenesis has been hindered by the lack of reliable genetic methods [21]. The availability of clone and protein resources would enable functional proteomics studies directed at the detection, prevention and treatment of this disease agent. Comprehensive studies using recombinant proteins can be used both to determine which proteins stimulate cell-mediated and humoral immune responses in the blood of infected individuals and to test the proteins in the *reverse vaccinology* approach [22]. Using HT pipelines described here, we constructed the first comprehensive sequence verified gene collection of *F. tularensis* (subsp*. tularensis SCHU S4*) and a corresponding *E. coli* expressed protein collection. The strategies adopted in generating these resources, as well as some of the challenges overcome in the completion of the collection, provide a guideline for HT gene cloning efforts.

## Results

### Generation of the *F. tularensis* gene collection

#### Cloning Strategy

The assembly of this protein coding clone collection was initiated by acquiring annotated genome sequence information to predict the relevant ORFs and design the PCR amplification primers. We started with a genome annotation kindly provided by H. Tettelin at the Institute for Genomic Research (TIGR) that predicted 2,036 ORFs based on a draft genome sequence provided by H. Svenson and P. Larsson. However, during the course of this project, a revised annotation of the genome sequence was published [20] that reduced the number of ORFs to 1804 with 1603 protein coding genes and adjusted many ORF boundaries from the earlier draft. The mapping and comparison of ORFs from preliminary and published genome annotations can be found in Table S1. Thus, this cloning project was accomplished in two phases corresponding to these two annotations. The annotated ORF information was parsed and imported into our Full-Length Expression Gene (FLEXGene) database [2]. Relevant features stored in our database for each ORF include CDS sequence; genome location; CDS length; GC content; NCBI protein GI number; and FTT number (an organism-specific identifier). Additionally, a new and unique tracking identifier was assigned to each ORF by FLEXGene.

**Figure 1:**
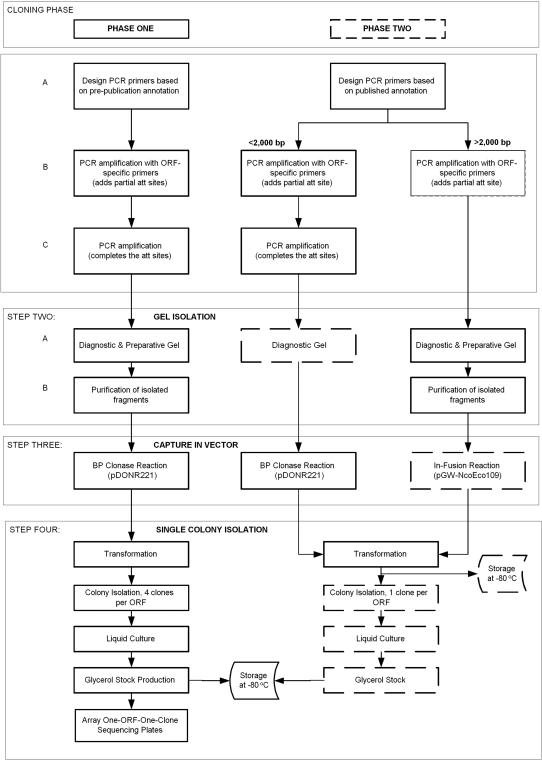
Schematic representation of the work flow used in genome cloning of *Francisella tularensis.* The entire process, from design of primers to production of clonal glycerol stocks, is shown. Most steps are common to both phases; steps specific to phase 2 are shown with a dashed line. The process began with design of primers for each ORF in the genome (Step 1A). The primers were used to amplify ORFs from genomic DNA (Step 1B). Subsequent amplification with universal primers (Step 1C) generated ORF sequences flanked by complete recombinational cloning sites for capture by BP (Step 3). For amplicons captured by In-Fusion in Phase 2, universal primed PCR was not necessary (Step 2B) as the capture reaction completes creation of the recombinational cloning sites. Successful PCR was monitored by agarose gel electrophoresis (Steps 2A and B). In Phase 1 all products were purified from preparative gels (Step 2B) and cloned into a recombinational cloning vector via the BP clonase reaction (Steps 3), whereas in Phase 2, the capture method depended on ORF size as indicated, with only diagnostic gels needed for short amplicons (Step 2A) and preparative gels needed when In-Fusion capture was performed. Competent bacteria were transformed with the reaction mix to yield colonies which were isolated robotically, cultured in liquid media and stored as 15% glycerol stocks (Step 4).

The cloning work-flow, shown in Figure 1, distinguishes between two phases of clone production. All barcoded plates and individual samples were tracked by FLEXGene, which also stores associated results such as culture growth, colony counts and electrophoretic gel images. In addition, each clone is associated with its target (reference) sequence for comparison during sequence validation.

In phase 1, all ORFs were amplified using gene specific primers with partial *att* sites to generate a primary PCR product, which was further amplified using universal primers to complete the *attB* recombinational cloning sites (Figure 1). The final PCR product was gel purified and subsequently cloned into an Entry vector, pDONR221 (Invitrogen), to generate a master clone. New primers were designed for all targeted ORFs in phase 2, which were channeled into two groups. For ORFs below 2,000 bp we employed the phase 1 strategy. For ORFs above 2,000 bp, we omitted the second PCR reaction and instead gel purified the amplicons with partial *att* sites for capture reactions using In-Fusion™ enzyme (Clontech) into a purified, linear pDONR221 derivative, pGW-NcoEco109. The full *attB* sites are regenerated as part of the In-Fusion™ capture step. Capture reactions were transformed into competent bacteria and either four colonies (phase 1) or one colony (phase 2) per gene were isolated robotically, stored as a 15% glycerol stock, and subjected to sequence verification.

#### Sequencing Strategy

In all cases, sequencing began with universally primed end reads for one isolate per gene and proceeded to internal reads where indicated (i.e., incomplete coverage but end reads acceptable). In cases where the first isolate failed during sequence validation, additional isolates were tested either by selecting another isolate (phase 1) or by re-plating the stored transformation mix for new colonies (phase 2). In cases where these strategies failed, cloning was repeated *de novo*. In both phases, we used our Automated Clone Evaluation (ACE) software to assemble the end reads and determine if they were sufficient to obtain complete sequence coverage (coding plus linker sequences). If coverage was complete, the assembled sequence was compared with the reference sequence to determine if the clone were acceptable. Discrepancies that occur in regions of low sequence confidence (typically phred<25) likely reflect sequencing errors, whereas discrepancies that occur in regions of high sequence confidence likely reflect true differences between the clone and its reference sequence. Clones were accepted if they had no high confidence discrepancies leading to protein truncations or frame shifts, no discrepancies in the critical linker regions, and no more than two amino acid differences with the reference polypeptide. If the coverage was not complete or if there were regions of unacceptably low confidence sequence, additional sequencing was performed. This process was repeated iteratively until all clones were either accepted or rejected.

#### Cloning and Sequencing Results

At least one isolate was obtained for more than 99% of the targeted 2,036 ORFs in phase 1. Sequence analysis revealed that 1500 ORFs were acceptable (73.6%; Table 1) based on the draft annotation. Subsequent to this analysis, a revised genome sequence of *F. tularensis* (subsp*. tularensis SCHU S4*) was published and further annotated at NCBI to predict 1,603 protein-coding genes [20]. A total of 1,104 ORFs were identical between the two annotations, of which we had successfully cloned 900. The other 499 ORFs were either new or adjusted ORFs, both warranting a new cloning attempt. Thus, the second phase focused on the 499 adjusted ORFs plus 204 ORFs that failed in the first phase (a total of 703 ORFs). Phase 2 was very successful, resulting in 696 acceptable clones for 634 ORFs (>97% for phase 2; Table 1). Overall, a total of 1,534 acceptable protein-coding clones matching the current NCBI annotation were obtained (96% success rate relative to the updated annotation). A complete list of these clones including their GenBank Accession Numbers and sequences can be found in Table S2 and at http://plasmid.hms.harvard.edu. Protein expression clones were generated via recombinational sub-cloning into the amino terminal hexa-histidine tag expression vector pDEST17 (Invitrogen), using similar automated pipelines, attaining a 97% first pass efficiency.

**Table 1:** Summary of the cloning process of two annotations of *F. tularensis*

	Phase1	Phase2
ORF Target	2036	703
Average ORF size (bp)	798 (range 90–4,269)	1025 (range 105–4,269)
Genome annotation	TIGR preliminary annotation (Feb 2004)	NCBI (Feb 2006)
Primer synthesis organization	Illumina	IDT
PCR polymerase	KOD	Phusion
Accuracy of polymerase (errors/bp)	1/290,000	1/770,000
Capture reaction	BP	Small gene: BP
		Large gene: InFusion
Isolate picking	4 per ORF	1 per ORF
Sequencing vector	pDONR221	pDONR221 & pDEST-17
PCR success rate	100%	100%
Capture success rate	99.2%	99.1%
**Clones for sequence validation**	**2852**	**987**
Number of reads	5835	3458
**Average number of reads per clone**	**2±1.7**	**3.5±3.6**
**Mutation rate (errors/bp)**	**1/608**	**1/3939**
Clones with linker changes	182 (6.4%)	6 (0.6%)
Clones with frameshift	239 (8.4%)	52 (5.3%)
Clones with inframe ins/del	7 (0.2%)	0
Clone with truncation mutation	84 (2.9%)	1 (0.1%)
Clone with> = 3 missense	67(2.3%)	3 (0.3%)
Clone with LQ discrepancy or unassembled (not further pursued)	768	229
**Number of clones accepted (includes redundant clones)**	**1505**	**696**
**Number of clones needed to finish a gene**	**1.9**	**1.6**
Clones match perfectly with reference	626 (21.9%)	663 (95.3%)
Clone with silent only	143 (5.0%)	7 (1.0%)
Clone with< = 2 mis-sense	736 (25.8%)	26 (3.7%)
**Accepted ORFs matching old annotation**	**1500**	**N/A**
**Accepted ORFs matching current NCBI annotation**	**900**	**634**
**Acceptance rate (current NCBI annotation)**	**81.5%**	**90.2%**

### Automated high-throughput production of *Francisella tularensis* proteins

#### Construction of an automated 96-well protein purification pipeline

The availability of isolated proteins for genes in *F. tularensis* enables proteome scale investigation into the role that each component of this microbe plays in cellular and humoral immunity. We established an automated workstation for HT production, isolation and analysis of microgram quantities of protein, sufficient for use in immunoassays (e.g., ELISPOT). Purification was based on immobilized metal affinity chromatography (IMAC) using magnetic beads. Microfluidic-based protein analysis of aliquots was performed to determine the relative purity and quantity of the each recombinant protein.

Automated analysis via Labchip90 provided a computerized, quantitative output of polypeptide size, concentration and purity for each protein. In order to separate information about the protein of interest from the other contaminating host cell proteins, we parsed the digital output files using in-house software that collected information about each protein peak into a tab-delimited format that could be uploaded into a database. This enabled queries to look for protein peaks of the expected molecular weight and, if found, to obtain key parameters (size, concentration and purity).

### Expression and affinity purification of 6xHIS tagged *F. tularensis* proteins

**Figure 2:**
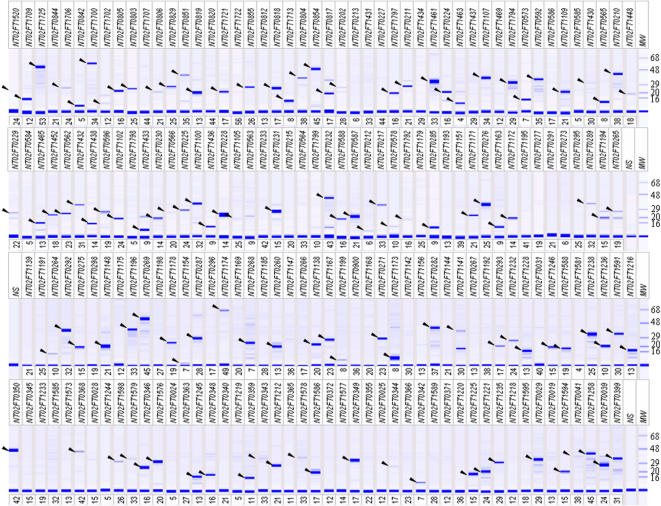
Representative virtual protein analysis gel of 188 proteins produced via the high-throughput protein production pipeline. The label NTFT02#### indicates the unique identifier for each ORF of *Francisella tularensis*. The expected molecular weights (based on predicted protein coding sequences of the ORFs) are shown below each lane. Black arrows (left side) indicate protein bands observed at approximately the expected molecular weight. MW, molecular weight; NS, no sample.

From 1961 sequence-verified full-length clones representing 96% of protein coding *F. tularensis* ORFs, we successfully expressed and purified 72% of the proteins. Protein isolation was considered successful when at least 120 ng of the putative recombinant protein was present and the apparent molecular weight was within +/− 40% of the theoretical molecular weight. Representative virtual gels of protein analysis generated from the pipeline are shown in Figure 2 and detailed information about all successfully purified proteins can be found in Table S3. Amongst the successful proteins, the concentrations varied from under 200 to more than 9,000 ng/µl with an average of 1494 ng/µl (SDEV: 7425 ng/µl). There was no apparent effect of protein size on the purification success rate for this collection, but the overall success rate for proteins predicted to contain trans-membrane domains (http://www.cbs.dtu.dk/services/TMHMM/) was only 57% compared with the overall success rate of 72%.

## Discussion

The genome sequence of *F. tularensis* was recently published and provides a starting point to explore new avenues of research, which should ultimately aid in the development of more effective vaccines and foster our understanding of tularemia. In this work, we present the production of a high quality clone collection for *F. tularensis* (subsp*. tularensis SCHU S4*) with general utility for a broad range of different protein-based assays. To accelerate progress towards that goal, we developed HT protein purification pipelines to produce proteins useful for immunological assays.

One of the major bottlenecks we encountered during the cloning of the *F. tularensis* ORFeome was a significant change to the preliminary genome annotation. We began our work using the best available annotation and when the revised annotation of the genome was released, we decided to incorporate this updated information into our production pipelines. Nearly 45% of the ORFs from our initial target set had to be classified as incorrectly annotated ORFs, with the majority being partial ORFs. The new annotation had two distinct effects on our pipeline. First, as our clone and protein productions were carried out simultaneously, downstream pipeline steps were burdened with some partial-length forms of genes along with full-length forms. Second, as the target set was revised, completion of clone production and protein purification took longer than initially expected. Both problems could be tolerated when balanced by an ultimately more complete and accurate clone collection.

Other factors that affected cloning success included the source of amplification primers and the use of polymerases with different fidelities (see Table 1). Changing the source for gene-specific primers in phase 2 decreased significantly the fraction of errors in the linker region (reduced from 6% to 0.6%). In addition, using a polymerase with higher fidelity, combined with improved primer quality, led to a 6 fold decrease in the overall rate of mutation from 1/608 to 1/3,939 base pairs, and decreased the number of attempted clones per final clone from 1.9 to 1.6. The second phase did require an increased number of sequence reads per clone; although it should be noted that this phase had an increased number of long or challenging clones (all ORFs that had failed the first phase were included again in the second phase).

For protein production, we relied on a widely used heterologous bacterial expression system, optimized experimental procedures, and developed a completely automated platform. The simplicity of the bacterial system facilitated automation in a way that may not have readily been possible using other protein production systems. Moreover, we were able to use the bacterial system to produce proteins of the required concentration and purity to perform immuno assays for over 72% of the proteins in the *F. tularensis* proteome. Failure in protein production cannot be attributed to nonsense or other mutations in the clone set, as each clone in the collection was fully sequenced and clones with any form of truncation were not included in the collection. What we categorized as ‘failure’ of the remaining 28% of the *F. tularensis* proteome is attributable at least in part to yields below our cut-off of 120 ng. Low expression may reflect inherent properties of the proteins (such as high hydrophobic content) that could lead to poor expression in or toxicity to the heterologous host cells. Finally, the conditions used for HT protein expression were by necessity optimized for a wide range of proteins. The use of individualized conditions might result in improved results for particular proteins. The protein expression clones generated in this study can readily be used to scale up protein expression to achieve higher amounts of protein if required. The use of the Gateway recombinational cloning system to build the clones further facilitates easy shuttling of ORFs into a variety of protein expression vectors with different affinity tags. As this collection includes a normalized stop codon at the end of the ORF, this collection is restricted to adding tags at only the N-terminus.

Automated protein analysis obviated the need to use gel-based chromatography and resulted in a quantitative digital output of protein concentration and purity. This form of output allowed normalization of protein concentration prior to use in downstream assays. Because our past experience suggested that HT experimentation could be error prone, we took precautions at every step to minimize manual intervention and ensure efficient tracking at the plate and sample levels.

In summary, the first complete full length sequence verified clone set representing the genome of *F. tularensis* (subsp. *tularensis SCHU S4*) was created. The clone collection was successfully used to generate a protein expression clone resource, which was subsequently used to produce proteins for over 72% of the *F. tularensis* proteome. The entire operation was automated and was supported by a LIMS, as well as custom databases and software tools. The clone repository serves as an important resource with which to probe the biology of *Francisella* and with slight alterations, the automated pipelines we developed will be used for a variety of different high-throughput assays. The clones generated in this study are openly available at http://plasmid.hms.harvard.edu. We expect that the operational methods adopted in this study will serve as an example for the design of similar processes relevant to other experimental systems.

## Materials and Methods

### Oligonucleotide design and ORF amplification

The ORFs were amplified using two consecutive rounds of PCR amplification from the genomic DNA of *Francisella tularensis Schu4*. In the first round, matched 5′ and 3′ oligonucleotides containing gene-specific sequences with normalized start (ATG) and stop (TAG) codons plus a short segment of the *attB1* and *attB2* sequences, respectively, was used to amplify each target ORF. The resulting product was then further amplified in the second round of PCR using “universal” primers that overlapped and completed the *attB1* and *attB2* sequences. This two-step PCR improves fidelity and lowers cost. Oligonucleotides were automatically designed using in-house software employing the nearest-neighbor algorithm to generate primer pairs that match the ends of the coding sequences with a specified melting temperature and then appends the partial *attB* tails as follows: forward primer, 5′TACAAAAAAGCAGGCTCCACC- atg→*gene-specific sequences*; reverse primer, 5′GTACAAGAAAGCTGGGTC -tag → *gene-specific sequences* (underlining indicates partial *attB* sequences). Second-step PCR universal primers were synthesized as follows: forward primer, 5′-GGGGACAAGTTTGTACAAAAAAGCAGGCTCC; reverse primer, 5′-GGGGACCACTTTGTACAAGAAAGCTGGGTC (underlined are *attB* sequences).

For phase 1, first and second-step PCR amplifications were done with KOD enzyme (Novagen). Conditions were as follows: PCR-1: 0.06 mM each primer, 1× KOD buffer 1, 0.2 mM dNTPs, 1 mM MgSO4, 1× KOD buffer 2, 200 ng genomic DNA, 0.6U KOD polymerase; 94°C 2 min, 15 cycles [94°C 15 s, 59°C 1 min, 68°C 6 min] 68°C 12 min, 4°C hold; PCR-2: 0.125 mM each *att*-primer, 1× KOD buffer1, 0.3 mM dNTPs, 1 mM MgSO_4_, 1× KOD buffer2, 0.6U KOD polymerase, 40% (v/v) PCR-1; 94°C 2 min, 6 cycles [94°C 15 s, 59°C 1 min, 68°C 6 min] 68°C 12 min, 4°C hold.

For phase two, both PCRs used Phusion™ enzyme (New England Biolabs) using the following conditions: PCR-1: 0.1 mM each primer, 1× reaction buffer, 0.2 mM dNTPs, 200 ng genomic DNA, 0.8U Phusion polymerase, 6% (v/v) DMSO; 94°C 2 min, 15 cycles [94°C 15 s, 52°C 1 min, 68°C 5 min] 68°C 12 min, 4°C hold; PCR-2: 0.125 mM each *att*-primer, 1x reaction buffer, 0.2 mM dNTPs, 0.8U Phusion polymerase, 6%(v/v) DMSO, 40% (v/v) PCR-1; 94°C 2 min, 5 cycles [94°C 15 s, 52°C 1 min, 68°C 5 min] 68°C 12 min, 4°C hold.

### Cloning of the F. tularensis FLEXGene collection

Cloning of the *F. tularensis* FLEXGene collection was performed as described in Figure 1, and as published previously [2], [7]. Briefly, the PCR amplified ORFs were recombined to generate ‘entry’ clones, i.e. ORFs captured in an entry or initial cloning vector that facilitates sub-cloning of the ORFs into vectors appropriate for specific experimental approaches. *E. coli* strain DH5αT1 (Invitrogen) was used for propagation of the clones. Expression clones for protein production were generated from the entry clones in a one-step recombinational sub-cloning reaction into pDEST17 (N-terminal 6xHIS tag; Invitrogen) as described by the manufacturers protocols and elsewhere [2]. All entry and expression recombinant clones are stored as DNA and as bacterial glycerol stocks (15% v/v glycerol) of single colony-selected *E. coli* transformants.

### Protein expression

Cell growth, transformation, and protein purification were performed according to the protocols in our laboratories and are described elsewhere [14]. Briefly, BL21 star (DE3) pLysS (Invitrogen) transformants harboring the recombinant plasmids were grown at 37°C as 1 ml cultures in a 96-well block (Marsh Biomedical Products) to an OD_600_ of ∼0.7. The cultures were induced with 1 mM IPTG and grown on a 96-well plate shaker (Multitron) at a speed of 900 rpm. After allowing a post-induction growth for a period of 4 hrs, the cells were harvested at 4°C and stored at −80°C for further use.

### Automated 96-well protein purification

Protein purifications were performed in 96-well plates using a BiomekFx (Beckman Coulter) robotic liquid handler under conditions optimized in our laboratory. Cell lysis, protein binding and washes were optimized by modulating the number of pipetting cycles, the shaking speed and the volumes of various reagents. For 6xHIS denaturing affinity purification, the robotic deck was setup using 15 ml of lysis buffer I (100 mM NaH_2_PO_4_, 10 mM Tris, pH 8.0), 15 ml of lysis buffer II (100 mM NaH_2_PO_4_, 10 mM Tris, 6M Guanidine hydrochloride, 10 mM, 2-Mercaptoethanol, pH 8.0), 25 ml of wash buffer (100 mM NaH_2_PO_4_, 10 mM Tris, 8M Urea) and 15 ml of elution buffer (wash buffer with 500 mM imidazole) for each 96-well block. A 96-well Magnabot (Promega, #V8151) and compatible plates (Greiner Bio-one, #650101) were used for purification steps and the final eluate was collected in a fresh 96-well plate.

### 6xHIS affinity purification

The cell pellets were thawed at room temperature for 15 min, lysed in the presence of protease inhibitors in 100 µl lysis buffer I, robotically resuspended in a 96-well block and agitated at 900 rpm for 10 min (5 min in the clockwise direction and 5 min in the counterclockwise direction). Then, 10 µl of DNase mix (10 mg/ml. DNase from Sigma Aldrich in 900 mM MgCl_2_, 100 mM MnCl_2_) was added to the lysate followed by agitation at 900 rpm for 10 min. Next, 100 µl of lysis buffer II was added to create denaturing conditions. The cell lysate was allowed to bind to 30 µl of MagneHIS (Promega #V8565) with shaking at 900 rpm for 20 min (10 min clockwise, 10 min counterclockwise). The beads were separated using a magnabot (24-pin magnet) and the remaining lysate was robotically pipetted and discarded. The MagneHIS beads with bound protein were washed three times with wash buffer. Bead adherence to the walls during washing was prevented by shaking at 900 rpm for 2.5 min clockwise and then 2.5 min counterclockwise. The bound protein was either directly used in assays or eluted in 50 µl elution buffer (i.e., wash buffer with 500 mM imidazole).

### Automated 96-well protein analysis

Protein analysis was performed in a 96-well format using a capillary-based instrument, the LabChip90 (Caliper Sciences). Protein samples were resuspended in analysis buffer (Caliper Sciences), heated to 96°C for 5 min., cooled to room temperature and briefly centrifuged to collect the sample. Distilled water (35 µl) was added to each sample prior to analysis. The analysis chip (Caliper Sciences) was primed according to the manufacturer's instructions. The automated protein analysis generated three different forms of output: a chromatogram that shows migration time; a virtual gel that mimics a Coomassie stained gel; and a results table that includes the estimated size, quality and quantity of each peak. The LabChip90 analyzed 96 proteins at a time with analysis time of 40 seconds per sample. The output results were parsed and imported into our protein database. As the error range for the LC90 was +/− 20%, any bands corresponding to +/− 40% of expected size and above the 120 ng cutoff were annotated as the correct band. The computed results were reviewed manually, and in the case of small proteins (<14 kDa), the size criteria were expanded to +/− 60% due to the resolution of the instrument in this range.

## Supporting Information

Table S1 Genome annotation
(0.19 MB XLS)

Table S2 Complete clone list
(1.71 MB XLS)

Table S3 Protein expression data
(0.17 MB XLS)
